# Review on Research about Traditional Chinese Medicine in Cancer Stem Cell

**DOI:** 10.1155/2017/4505194

**Published:** 2017-10-04

**Authors:** Yaohan Wang, Li Feng, Bingkui Piao, Peitong Zhang

**Affiliations:** ^1^Department of Oncology, Guang'anmen Hospital, China Academy of Chinese Medicine Sciences, No. 5 Beixiange, Xicheng District, Beijing 100053, China; ^2^Traditional Chinese Medicine Department, National Cancer Center/Cancer Hospital, Chinese Academy of Medical Science and Peking Union Medical College, Beijing 100021, China

## Abstract

Cancer stem cells (CSCs) are small subpopulations of neoplastic cells within a tumor, which have self-renewal and differentiation abilities and could generate new tumors with few cells. Researches have showed that CSCs are considered the most likely reason for cancer recurrence and metastasis. Accumulating evidences have showed that traditional Chinese medicine (TCM) has significant effect on CSCs. It could inhibit the proliferation, self-renew, and multidifferentiation of CSCs. We aimed to summarize the theories of CSCs in TCM, the inhibitory effect, and the pathway on CSCs of TCM. This review will provide potential new strategies and alternative perspectives for CSCs treatments and basic research into complementary and alternative medicine.

## 1. Introduction

Local and distant recurrence of malignant tumors following radio- and/or chemotherapy correlates with poor prognosis of patients. Among the reasons for cancer recurrence, cancer stem cells (CSCs) are considered the most likely cause [1, 2]. Cancer stem cells (CSCs) are small subpopulations of neoplastic cells within a tumor, usually less than 5 percent, which are able to generate new tumors in appropriate animal hosts. These cells divide asymmetrically which on one hand yield CSCs and on the other hand differentiate into neoplastic cells forming tumor tissue [3–5]. It has been demonstrated that cancer stem cells derive from normal stem cells and differentiated somatic cells that undergo transformation and dedifferentiation, respectively, under certain conditions [1], which have self-renewal and differentiation abilities [6]. Additionally, the thought that CSCs are the cause of tumorigenesis, progression, invasion, metastasis, chemoradiotherapy resistance, and relapse makes their analysis more important [7, 8]. The American Association for Cancer Research (AACR) in 2006 sought to unify conceptions of this cell type. In their working definition, the AACR determined that a cancer stem cell is “a cell within a tumor that possesses the capacity to self-renew and to cause the heterogeneous lineages of cancer cells that comprise the tumor” [9, 10]. These cells had many similarities to stem cells, such as self-renewal, multidifferentiation [11–13], similar growth mechanism [14], and activity of telomerase [15], and both transfer to different tissues [16]. Traditional Chinese medicines (TCM) have drawn a great deal of attention in recent years for their potential in the treatment of cancer. And accumulating evidences have showed that traditional Chinese medicine has significant effect on CSCs. So it is necessary to further research the effect of TCM on CSCs.

## 2. TCM and Cancer Stem Cells

The theory of cancer stem cell is proposed in past decade, so there is no record about CSCs in ancient Chinese medicine. According to CSC's biological behaviors and effects on tumor metastasis or recurrence, 3 ideas had been put forward about CSCs in TCM.

### 2.1. Essence Deficiency Had a Close Relationship with CSCs

Stem cells could differentiate into normal cells which are basic components in life. In ancient Chinese medicine, the records of CSCs were included in the theories of Essence, Five Elements, and Visceral Manifestation [17–19]. Qi is the basic component in body, which participates and maintains activities in life. Essence deriving from the kidney could promote growth of body as the power of life. Essence, which is the origin of all things in the universe, is the power of the qi in life and is indispensable in life activities. It is similar to stem cells, so we classify stem cells as Essence of kidney in TCM [20]. Under the physiological condition, Essence of kidney plays an important role in life activities. When Essence of kidney is insufficient, the tumor occurs and metastasizes. CSCs which have the abilities of self-renewal, differentiation, and oncogenicity are derived from mutant stem cells and related to tumor occurrence and metastases. Accordingly, Essence of kidney deficiency had a close relationship with CSC.

### 2.2. Imbalance of Ying and Yang Could Induce CSCs

Researches have shown that the cause of oncogenesis may relate to CSCs regulate by the niche where they reside. Now few treatments have targeted cancer stem cells. The factors in the niche, controlling CSCs, are similar to the characteristics of yin-yang in traditional Chinese medicine. Under physiological conditions, regulated factors in the niche controlling oncogenes and suppressor genes, proliferation, differentiation and apoptosis, and activation and inactivation of signal transduction pathways are maintained in a balanced condition in order to regulate the normal quantity of stem cells. It is similar to the function of yin and yang in TCM theory; for example, the oncogenes resemble yang, and suppressor genes resemble yin. Once the balance of oncogenes (yang) and suppressor genes (yin) is destroyed, the proliferation of CSC would be out of control, cancer would occur, and relapse and metastasis would develop. Therefore, it is important to suppress oncogenes (yang) or activate suppressor genes (yin) to control the proliferation of CSC [21]. So, the treatment on the regulation of the niche of CSC is quite necessary according the principle of yin-yang in TCM [21].

### 2.3. Phlegm Might Be One of the Causes to Induce CSCs

Usually, the lump is pigeonholed as phlegm in TCM. Zhu Danxi thought that most of lumps in body were induced by “phlegm,” and the phlegm belonged to tangible phlegm. Though CSCs were fewer in total tumor cells, they were the most important factors to induce tumor occurrence and metastasis. So, from this point, CSC was similar to tangible phlegm. Intangible phlegm, which was another type of phlegm, could arrive to all organs with qi moving. Zhu Danxi thought intangible phlegm was harmful to health, because of moving everywhere, from head to toe. And CSCs have lower ability of adhesion and cell polarity and higher ability of movement than tumor cells. So intangible phlegm had more close relationship with CSC [22, 23].

## 3. Effects and Mechanisms of TCM on CSCs

Accumulating evidences had revealed the existence of CSCs in the majority of leukemia cases and a number of solid tumors, including colorectal cancer [24–27].The stem cell-like characteristics gave CSCs the capacities for continuous self-renewal, multidifferentiation, and natural drug resistance, resulting in cancer relapse and metastasis and leading to the eventual failure of clinical anticancer treatment [28, 29]. Therefore, discovering novel agents that target CSCs has the potential to improve the effectiveness of anticancer treatment.

Traditional Chinese medicines have gained great attention as certain naturally occurring compounds have been demonstrated to possess anti-CSC abilities [30, 31]. The research in animals has proved that curcumin and quinacrine liposomes not only could enhance apoptosis effects and endocytic effects of C6 glioma stem cells in vitro, but also could increase the survival period of brain glioma-bearing mice and inhibit the growth of gliomas in vivo [32]. The preclinical researches about influence of TCM on CSCs have found that traditional Chinese medicine could affect CSCs on stages in the growth of CSCs; for example, resveratrol can inhibit the cancer cells form spheres at the formation of CSCs [33] and sulforaphane has an inhibitory effect on biological behaviors, which are the important features of CSCs, such as self-renewal, proliferation, and differentiation [34–36], and some traditional Chinese medicines not only inhibit the behaviors of CSCs, but also could reverse drug resistance and promote autophagy. Therefore, it is necessary to research the effects and mechanisms of TCM on CSCs to help us find the new target of cancer therapy in clinic in future.

### 3.1. TCM Has Effect on CSCs by Inhibiting CSCs Viability

*Hedyotis diffusa* Willd was a drug to treat cancer in TCM, with the heat-clearing, detoxicating, and antitumor effect. The ethanol extract of* Hedyotis diffusa* Willd [30] could inhibit colon cancer stem cells sphere formation, differentiation in vitro by downregulating the *β*-catenin and TCF4 mRNA, and inhibiting *β*-catenin/Wnt pathway and CK20 and CD133 mRNA in colon cancer stem cells, which was time-dose dependent. In addition, the researches had showed that bufalin which was the main component of Chansu could inhibit tumor cells proliferation [31] and promote apoptosis [37–39]. Yeh et al. [40] found that bufalin inhibited the stemness of osteosarcoma cancer stem cells. Moreover, the research showed that miR-148a might be a target of bufalin, and miR-148a downregulated DNMT1 and p27 to inhibit the stemness of OS cells. Honokiol (HNK) could inhibit sphere formation and stemness in melanoma cells. It significantly decreased the number and size of microspheres in a dose dependent manner. And western blot showed that HNK significantly enhanced phosphorylation of AMPK in melanoma cells [41].

### 3.2. TCM Has Effect on CSCs by Inducing CSCs Apoptosis

CD133 was a marker in lung cancer stem cells. CD133 cells in A549 were fewer after treatment by nanorealgar. Nanorealgar could induce both A549 cells and A549 cancer stem cells apoptosis and reduce the number of cancer stem cells in a dose dependent manner by caspase dependent mitochondrial pathway, but the sensitivity of cancer stem cells to nanorealgar was much lower than the A549 cells [34]. Sulforaphane induced prostatic cancer stem cells apoptosis by inducing caspase-3 and dephosphorylation FKHR, and it also could upregulate caspase-3, PARP, and BAX and downregulate Bcl-2 to induce chronic leukemia stem cells apoptosis [42].

TCM formulas had the same effect on CSCs. Sijunzitang serum could upregulate Bax and downregulate Bcl-2 to induce side population cells apoptosis and suppress proliferation in gastric cancer [43]. And TCM formulas combined with chemotherapy induced lung cancer stem cells apoptosis, because of the herbs in formulas which had qi-benefiting and Essence-nourishing effect and suppressed Wnt pathway [44].

### 3.3. TCM Could Arrest CSCs Cell Cycle at G0/G1

Evodiamine was a component of a traditional Chinese medicine, which selectively targets CSCs of breast cancer at a concentration that does show a little or no cytotoxic effect on bulk cancer cells. Evodiamine could make bulk cancer cells accumulate at the G2/M phase [45], while it selectively killed CSCs instead, not arresting CSCs in a specific cell cycle phase. In a high concentration, evodiamine kills CSCs and bulk cancer cells in the same mechanism, and in a low concentration, evodiamine could selectively activate p53 and p21 and decrease inactive Rb, the master molecules in G1/S checkpoint, which is more efficient in CSCs than bulk cancer cells. These data collectively suggested a novel mechanism involving CSCs-specific targeting by evodiamine and its possible use in the therapy of breast cancer [46]. Kan et al. formulas arrested nasopharyngeal carcinoma stem cells at G0/G1 and downregulated the CSCs marker CD44 and ABCG2 and induced CSCs apoptosis to kill cancer stem cells [47].

### 3.4. TCM Could Downregulate CSCs Markers

Cancer stem cell markers usually were divided into two parts: (1) the markers only expressed in cancer stem cells, such as CD133; (2) the markers expressed in both cancer stem cells and embryonic stem cell, such as Sox-2, Oct-4, and Nanog. These markers not only identified CSCs, but also affected the formation of CSCs.

Curcumin, a component of Jianghuang, could significantly inhibit sphere formation and dephosphorylation STAT3 [48], and water-soluble curcumin downregulated CD44+ and CD24+ by injection [49]. Sulforaphane (SNF) could inhibit SHh pathway, which targeted downregulating downstream Nanog, Oct4, VEGF, and PDGFR and inhibited pancreatic cancer stem cells ability of self-renewal [50]. Resveratrol not only induced apoptosis to inhibit CSCs self-renewal, but also could downregulate Sox-2, Oct-4, Nanog, c-Myc, and ABCG2. So, resveratrol could significantly inhibit CSCs metastasis and EMT [51].

### 3.5. TCM Could Sensitize CSCs to Chemotherapeutic Drugs by Reversing Drug Resistance

Mitomycin (MMC) or curcumin alone marginally reduced the BCSC population in mammospheres; however, together, they reduced the BCSC population in CD44+CD24−/low cells by more than 75%. Curcumin [52] sensitized breast cancer cells to chemotherapeutic drugs by reducing the BCSCs population mainly through a reduction in the expression of ABCG2 and ABCC1. It had demonstrated that fumitremorgin C, a selective ABCG2 inhibitor, reduced BCSCs survival to a similar degree as curcumin did. In addition, curcumin improved the sensitivity of paclitaxel, cisplatin, and doxorubicin in breast cancer cell lines MCF-7 and MDA-MB231, as shown by more than 2-fold decrease in the half-maximal inhibitory concentration of these chemotherapeutic agents.

Pien Tze Huang (PZH), a well-known TCM formula that was first prescribed more than 450 years ago in the Ming Dynasty, had been used in China and Southeast Asia for centuries as a folk remedy for various types of cancer [53–55]. In addition, PZH could inhibit CSCs. And PZH significantly reduced the percentage and the viability and sphere-forming capacity of the colorectal cancer stem-like SP cells in a dose dependent manner, which indicated that PZH had ability of suppressing the growth of colorectal cancer stem cells. Moreover, PZH markedly downregulated the mRNA levels of ABCG1 and ABCG2, which were members of the ABC transporter superfamily and contributed to the SP phenotype and multidrug resistance. The dates suggested that inhibiting the growth of CSCs is a potential mechanism by which PZH could be used in cancer treatment [56].

### 3.6. TCM Could Promote CSCs Autophagy

Autophagy was normally a degradative mechanism for the removal of tumor of bulk cytoplasmic constituents through the endosomal/lysosomal system [30, 57, 58]. Previous studies revealed that autophagy was involved in the cell death induced by anticancer drugs such as 5-fluorouracil and rapamycin [59, 60]. And ginsenoside F2 (F2), a component of Renshen, was assessed for its antiproliferative activity against breast cancer stem cells (CSCs). F2 could induce the formation of acidic vesicular organelles, recruitment of GFP-LC3-II to autophagosomes, and elevation of Atg-7 levels, suggesting that F2 initiates an autophagic progression in breast CSCs [61].

### 3.7. TCM Could Inhibit CSCs Invasion

Brain tumor stem cells had played an important role in tumor occurrence and invasion. It had a close relationship with tumor invasion and prognosis [62]. The traditional Chinese medicine for promoting blood circulation and removing blood stasis could upregulate TIMP1 mRNA to enhance the inhibitory effect of matrix metalloproteinase (MMPs) to inhibit invasion of BTSCs in vitro. The extracellular matrix (ECM) plays the role of a barrier in glioma cell invasion, and MMPs could degrade ECM and remove obstacle of glioma cells, leading to tumor invasion and metastasis. The dates showed that overexpression of TIMP could make MMP inactive by combining with it to inhibit tumor invasion [63].

The effects and mechanisms of traditional Chinese medicine or extracts of TCM on CSCs were listed in Table 1.

## 4. The Pathway about CSCs in TCM

Cancer stem cells derived from mutant stem cells, so the pathway which affected stem cells also could affect CSCs [35, 64, 65], such as Wnt, Hedgehog, and Notch; meanwhile some new pathways were found in further research on CSCs. Now we only introduce the pathways about CSC treated by TCM.

### 4.1. Notch-1 Pathway

Ponnurangam [67] had showed that the combination of honokiol and IR reduced numbers and size of spheroids, which was coupled with reduced expression of CSC marker protein DCLK1. In addition, there were reduced levels of activated Notch-1, its ligand Jagged-1, and the downstream target gene Hes-1. Furthermore, expression of components of Notch-1 activating *γ*-secretase complex (presenilin 1, nicastrin, Pen2, and APH-1) was also suppressed. On the other hand, the honokiol effects were mitigated when the Notch intracellular domain was expressed. In addition, the honokiol-IR combination reduced levels of DCLK1 and the Notch signaling-related proteins in the xenograft tissues. Together, these data suggested that honokiol was a potent inhibitor of colon cancer growth that targets the stem cells by inhibiting the *γ*-secretase complex and the Notch signaling pathway. Zhang [66] had found that Xiaotansanjie could inhibit CSCs proliferation, the expression of VEGF, angiogenesis, and microvessel density (MVD) in a dose dependent manner in vitro. And further research showed that Xiaotansanjie decoction inhibits CSC proliferation and angiogenesis was related to the downregulation of Notch-1 and Hes1.

### 4.2. Wnt/*β*-Catenin Pathway

Honokiol exerted many anticancer effects on various types of cancer cells [68]. The research explored its effects on the elimination of cancer stem-like side population (SP) cells. Honokiol did dependently reduce the proportion and induced apoptosis of SP. And it inhibited the CD44 and Wnt/*β*-catenin pathway of SP cells. The Wnt signaling transducers, such as *β*-catenin and TCF-4, were decreased in honokiol-treated SP cells, while the *β*-catenin degradation promoting kinase GSK-3 *α*/*β* was increased. Consistently, the protein levels of *β*-catenin downstream targets such as c-Myc and cyclin D1 were also downregulated. Furthermore, the *β*-catenin-related EMT markers such as Slug and Snail were markedly suppressed by honokiol. The findings indicated honokiol might be able to eliminate cancer stem cells through apoptosis induction, suppression of Wnt/*β*-catenin pathway, and inhibition of EMT [69]. Wnt/*β*-catenin could also affect cancer stem cells differentiation and viability in colon cancer stem cells.* Hedyotis diffusa* Willd inhibited *β*-catenin and TCF4 gene transcription in Wnt/*β*-catenin pathway, suppressed the activity of Wnt/*β*-catenin, which inhibited the transcription of gene CK20 and gene CD133, and affected CSC differentiation and viability [30]. Sulforaphane could inhibit breast cancer stem cell growth to 80% and the microsphere formation to 75% at the concentration of 5 mol/L. It was demonstrated that sulforaphane downregulated Wnt/*β*-catenin to inhibit ALDH+ cells proliferation in vivo [70].

### 4.3. Hedgehog Pathway

The Hedgehog (Hh) signaling pathway controls tissue polarity, patterning maintenance, and stem cell maintenance during embryonic development [86].The recent researches find that Hh pathway has close relationship with CSCs self-renewal. Lu et al. [87] found that* Scutellaria barbata* could suppress the cell viability and ability of self-renewal and downregulate the colon cancer stem cells markers CD133 and Lgr5 mRNA in dose dependent manner. The inhibitory effect at 500 *μ*g/ml of* Scutellaria barbata* is the same as GANT61 which is the inhibitor of Hedgehog pathway at 10 *μ*g/m. Further research shows that* Scutellaria barbata* could significantly suppress Ptch1 mRNA and Gli1 mRNA in Hedgehog pathway. When Hedgehog (mainly SHh) binds with the Ptch receptor (mainly Ptch1), the Hedgehog signaling pathway is activated. At this time, Smo inhibitor which is released by active Pth releases signaling cascade to make Gli (especially Gli1) be activated and transfer into the nucleus to make target gene transfer, such as Ptch, Gli, and Hip (Hip1) which inhibit Hedgehog disperse. That leads to CSC self-renewal [88].

### 4.4. Rb-p21-p53 Pathway

Evodiamine significantly increased the proportion of CSCs at the G1 stage and caused a massive volume of dying cells in a dose dependent manner, while the bulk cells were arrested at G2/M but not serious cell death yet. The mechanism showed that the decrease of expression was seen in phosphor-Rb (Ser795 and Ser 807/811), the master gene at the G1/S transition. Additionally, cyclin B1 and Fox M1, a key cyclin and key transcription factor at the G2/M stages, respectively, significantly diminished. The DNA breakage surveillance protein Chk1 and the TYRO3 which was required for G1/S transition in breast cancer cell decreased as well. p21, another key cell cycle blocker at G1/S, was found to increase the most by evodiamine. And the RPPA results showed that p53 and the phosphorylation of Akt were increased by evodiamine in CSLC, not in bulk cancer cells. Overall, p53 and p21 activation followed by Rb activation were the events that led to CSC death at the G1/S checkpoint [46].

### 4.5. *β*-Catenin/ABCG2 Pathway

Accumulating evidence has shown that *β*-catenin signaling in CSCs has a close relationship with chemoresistance and ABCG2 expression. Isoliquiritigenin (ISL), a compound of Gancao, had synergistic effects with chemotherapeutic drugs to inhibit breast cancer cell proliferation and colony formation. ISL could limit the SP and CSCs ratios by inhibiting self-renewal and multidifferentiation abilities in breast cancer. The research showed that ISL could activate the proteasome degradation pathway to inhibit *β*-catenin/ABCG2 signaling. GRP78 is the direct target of ISL. ISL inhibited ATPase activity by docking into the ATP domain of GRP78, which resulted in its dissociation from *β*-catenin. ISL could also enhance the sensibility of chemotherapy breast CSCs via the GRP78/*β*-catenin/ABCG2 pathway, with little toxicity in normal tissues and mammary stem cells. Therefore, the research has suggested that ISL could enhance breast CSCs chemosensitivity and highlight the significance of GRP78 in mediating cancer drug resistance and *β*-catenin signaling in CSCs [70].

### 4.6. PI3K/Akt/mTOR Pathway

The research about pancreatic cancer stem cells found that [79] rottlerin could reduce the viability of pancreatic cancer stem cells and induce apoptosis to form cytoplasm cavity and upregulate autophagy markers LC3-I, LC3-II, Atg7, and Beclin-1. Further research showed that phosphorylation of Akt and mTOR was inhibited to induce apoptosis, downregulate Bcl-2 and Bcl-X1, upregulate Bax, and activate caspase-3 and caspase-9 in CSCs treated by rottlerin. In addition, Akt inhibitor promoted CSC apoptosis induced by rottlerin. Silencing genes Atg7 and Beclin-1 or autophagosome inhibitor significantly inhibited apoptosis and autophagy induced by rottlerin. Therefore, rottlerin might induce CSC autophagy to apoptosis by inhibiting PI3K/Akt/mTOR pathway.

### 4.7. TGF-*β*/Smad Pathway

TGF-*β* could induce epithelial mesenchymal transition (EMT) and CSCs formation in breast cancer by releasing cross membrane signaling to induce Smad2/Smad3 to combine with Smad4 to activate Smad pathway, which could upregulate N-cad, VIM, and FN. Fuzheng Jiedu decoction significantly inhibited the expression of protein of EMT, such as E-cadherin, N-cadherin, and vimentin. Further research showed that Fuzheng Jiedu decoction might inhibit TGF-*β*mRNA and phosphorylation of Smad2/3 to inhibit TGF-*β*/Smad pathway and EMT to inhibit CSC formation [78].

### 4.8. AMPK Pathway

Honokiol (HNK) is a compound derived from Hopu, a plant that has been used in traditional Chinese medicine. Many researches showed HNK has antitumor activity with relatively low toxicity. HNK [42] could significantly inhibit the spheroid forming and stemness in melanoma cells. HNK significantly and dose-dependently reduced the number and size of melanospheres and the cellular ATP pool. The research suggested the AMPK pathway plays a central role in cellular energy homeostasis and acts as an energy sensor in cells. And further research showed that honokiol enhanced phosphorylation of AMPK to induce AMPK activation in melanoma cells.

Except for the signaling pathways mentioned above, there are others to affect the cancer stem cells; for example, FOXO3/LKB1/AMPK/PGC-1*β*/PDHA1 pathway is essential for CD44 expression and CSCs properties [89]; the mutual regulation between STAT3 and miR-181b could induce esophageal cancer stem-like cells proliferation and apoptosis resistance via CYLD pathway [90], and miR-34c-5p inhibits amphiregulin (AREG) to suppress ovarian cancer stemness and drug resistance via the AREG-EGFR-ERK pathway [91]. But whether TCM could via these pathways have effect on CSCs is not clear. In general, the factors which have a close relationship with CSCs are mainly epithelial mesenchymal transition (EMT) and Niche [92, 93]. So study of the pathways in EMT and Niche and influence of TCM on them may be the new way to CSCs therapy.

## 5. Conclusions

The mechanisms about cancer stem cells were complicated and varied. It was difficult to inhibit cancer stem cells only by targeting single pathway or mechanism. Traditional Chinese medicine was a huge goldmine and played an important role in the treatment of cancer stem cells. It had more advantages than western medicine, such as multiple targets in CSC treatment. So TCM combined with chemotherapy or radiotherapy had significant effects on CSC. The preclinical and clinical researches had demonstrated that TCM significantly inhibits tumor recurrence, metastasis, and prolonged survival [33, 71]. It was important to further study how TCM worked in CSC treatment and the effect of combining with chemotherapy or radiotherapy to improve the 5-year survival and the quality of life of cancer patients. However, the researches on CSC still had some problems: (1) The compounds of Chinese medicine were complex, especially, and had different biological function. Serum pharmacology was a common method of in vitro studies on TCM compound formulas [72], but the chemicals in TCM in serum are unstable and interfere with other factors. Therefore, serum pharmacology was not entirely accepted in TCM basic science. So new method about pharmacology is necessary. (2) TCM had a recorded history of over 2,000 years that may be used to guide modern treatments for disease and identify neglected but potentially useful treatment strategies [73]. However this process is based on ancient TCM theories of tradition and history that fail to take into account evidence-based medicine. An increasing number of clinical trials investigating a variety of TCM interventions have been registered in international trial registries, and the design of registered TCM trials has improved by using techniques such as sample size estimation, blinding, and placebos [36]. While the researches about CSC in TCM were few in clinic, so the high-level clinical evidence for CSC in TCM may be our target in future.

## Figures and Tables

**Table 1:** Effects and mechanisms of TCM or extracts on CSCs.

	Effects on CSCs	Mechanisms
Sulforaphane [64]	Inhibiting CSC self-renew	
Wusan granules [65], centralizer class of anticancer traditional Chinese medicine [35]	Inhibiting CSCs proliferation	
Xiantansanjie [66]	Inhibiting CSCs proliferation	Inhibiting Notch-1 pathway
Bufalin [40]	Inhibiting CSCs proliferation, differentiation, and sphere formation	miR-148a downregulated DNMT1 and p27
Sanguinarine [67]	Inhibiting CSC proliferation, invasion, and apoptosis	Inhibiting Wnt/*β*-catenin pathway
Ethanol extract of HDW [30, 66], sulforaphane [68]	Inhibiting sphere formation	Inhibiting Wnt/*β*-catenin pathway
Resveratrol [69], brucine [70]	Inhibiting sphere formation	
Honokiol [41]	Inhibiting sphere formation	Inducing AMPK phosphorylation
Curcumin [48], ursolic acid [33]	Inhibiting sphere formation	Inhibiting STAT3 phosphorylation
Ursolic acid [71]	Inhibiting proliferation and inducing apoptosis	Inhibiting EMT
Extract from *Sophora flavescens* [72], Feiyanning [44]	Inhibiting CSCs proliferation and inducing apoptosis	Inhibiting Wnt pathway
Pien Tze Huang [73]	Inhibiting the viability and promoting the apoptosis and differentiation	suppressing the Notch-1 pathway
Ginsenoside Rg3 [36], ginsenoside F2 [61], Huangqintang [74], Ligustrazine, tanshinone IIA [75]	Inducing CSC apoptosis	
Nanorealgar [34]	Inducing CSC apoptosis	Activating caspase-3
Fuzheng Jiedu, verticine, peiminine [76]	Inducing CSC apoptosis	Inhibiting EMT, downregulating TGF-*β*/Smad pathway
Sulforaphane [42]	Inducing CSC apoptosis	Activating caspase-3, inhibiting FKHR phosphorylation
Sijunzitang [43]	Inducing CSC apoptosis	Upregulating the expression of apoptosis protein BAX and downregulating Bcl-2
Rottlerin [77]	Inducing CSC apoptosis	Inhibiting PI3K/Akt/mTOR pathway and activating caspase in cascade
Evodiamine [46]	Arresting CSC cell cycle	Activating p53-p21-Rb pathway
Yiqijiedu [47]	Arresting CSC cell cycle, inhibiting the expression of CSC marker, and inducing apoptosis	
Jieduxiaozhengyin, Fuzhengyiliu [78], ginsenoside Rg3 [79], tetrandrine [80], curcumin [49]	Inhibiting the expression of CSC marker	
Resveratrol [52]	Inhibiting the expression of Nanog and Oct4	
Sulforaphane[50]	Inhibiting the expression of Nanog and Oct4	Inhibiting Shh pathway
Honokiol [67]	Inhibiting the expression of CD133	Inhibiting Notch-1 pathway
The traditional Chinese medicine for promoting blood circulation and removing blood stasis [63]	Inhibiting CSCs invasion	Inducing TIMP1 mRNA and inhibiting MMP
Ligustrazine [81, 82]	Inhibiting CSCs invasion	Inhibiting the expression of the integrin, CD44V6, VEGF, and HIF-1*α*
Isoliquiritigenin [70], Ligustrazine, tanshinone IIA [83]	Reversing drug resistance	Inhibiting *β*-catenin/ABCG2 pathway
Curcumin [52, 84], Jianpiyiwei [85], Pien Tze Huang [56]	Inhibiting the expression of ABCG2	
